# Radiomics-Based AI for the Diagnosis and Prognosis of Vessels Encapsulating Tumor Clusters in Hepatocellular Carcinoma: Systematic Review and Meta-Analysis

**DOI:** 10.2196/90931

**Published:** 2026-07-02

**Authors:** Xuefeng Hua, Rongdang Fu, Ziwei Yin

**Affiliations:** 1 Department of Hepatobiliary and Pancreatic Surgery, Guangzhou First People's Hospital, South China University of Technology, Guangzhou, Guangdong, China; 2 First People's Hospital of Foshan (Foshan Hospital affiliated to Southern University of Science and Technology), School of Medicine, Southern University of Science and Technology, Foshan, Guangdong, China; 3 Department of Oncology, Third Xiangya Hospital of Central South University, 138 Tongzipo Rd, Yuelu District, Changsha, Hunan, 410013, China, 86 17519983211

**Keywords:** radiomics, artificial intelligence, hepatocellular carcinoma, vessels encapsulating tumor clusters, meta-analysis, PRISMA, Preferred Reporting Items for Systematic reviews and Meta-Analyses

## Abstract

**Background:**

Vessels encapsulating tumor clusters (VETCs), a CD34-positive vascular pattern in hepatocellular carcinoma (HCC), are linked to aggressive biology, early recurrence, and poor survival. Because pathologic VETC assessment requires postoperative immunohistochemistry and may be affected by sampling, preoperative noninvasive prediction remains clinically important. Radiomics-based artificial intelligence (AI) applied to routine contrast-enhanced imaging may provide a surrogate marker, but evidence across has not been comprehensively appraised.

**Objective:**

This study aimed to evaluate the diagnostic accuracy and prognostic value of radiomics-based AI models for noninvasive VETC prediction in HCC using PICOTS (patient population, intervention, comparator, outcomes, timing, and setting) and PRISMA-DTA (Preferred Reporting Items for Systematic Reviews and Meta-Analyses for Diagnostic Test Accuracy) frameworks.

**Methods:**

We searched PubMed, Embase, Web of Science, and the Cochrane Library, gray literature, and citations (original search July 11, 2025; updated April 17, 2026). The original search was completed on July 11, 2025, and the reconstructed strategy was rerun on April 17, 2026. Eligible retrospective cohort studies developed or validated radiomics or deep-learning models using contrast-enhanced magnetic resonance imaging (CEMRI), contrast-enhanced computed tomography (CECT), contrast-enhanced ultrasound (CEUS), or 2-[¹⁸F]fluoro-2-deoxy-D-glucose positron emission tomography or computed tomography ([^18^F]FDG PET/CT) to predict CD34-confirmed VETC and reported 2×2 diagnostic data and/or hazard ratios (HRs) for early recurrence. Mutually exclusive cohorts were treated as separate datasets only when patient overlap was absent. Risk of bias was assessed with the Prediction model Risk Of Bias Assessment Tool+AI, and certainty with GRADE (Grading of Recommendations, Assessment, Development, and Evaluation). Diagnostic accuracy was synthesized with bivariate random-effects models; prognostic HRs were pooled with restricted maximum likelihood+Hartung-Knapp-Sidik-Jonkman random-effects models.

**Results:**

In total, 15 studies (729 internal-validation and 613 external-validation patients) were included; 14 were from China and 1 from Japan. Moreover, 10 studies used CEMRI, 3 CECT, 1 CEUS, and 1 [^18^F]FDG PET/CT. CEMRI-based AI showed the best performance: sensitivity=0.84 (95% CI 0.73-0.93; 95% prediction interval [PI] 0.45-1.00), specificity=0.79 (95% CI 0.70-0.86; 95% PI 0.50-0.97), and area under the curve=0.87. Meta-regression suggested that center type, validation design, algorithm class, and magnetic resonance imaging field strength contributed to specificity heterogeneity. CECT, CEUS, and [^18^F]FDG PET/CT evidence was limited. AI-predicted VETC positivity was associated with early recurrence (HR 2.34, 95% CI 1.93-2.84). GRADE certainty ranged from low to moderate, mainly due to imprecision, risk of bias, and heterogeneity.

**Conclusions:**

This review is innovative because it integrates diagnostic accuracy, modality comparison, algorithm performance, and recurrence prognosis for AI-based VETC prediction. Unlike previous modality-specific reviews, it clarifies what AI brings to the field: a potential preoperative bridge between imaging phenotypes and biologically aggressive HCC. Real-world use should remain cautious and decision-supportive, given retrospective designs, geographically concentrated cohorts, limited external validation, heterogeneity, risk of bias, and low-to-moderate GRADE certainty, rather than replacing histopathology or multidisciplinary clinical judgment in practice.

## Introduction

Hepatocellular carcinoma (HCC) is the most common primary malignant liver tumor, accounting for approximately 90% of liver cancer cases. According to the latest global statistics, liver cancer remains a major health burden with approximately 865,269 new cases and 757,948 deaths recorded in 2022. It is characterized by an age-standardized mortality rate of 7.4 per 100,000, making it the third leading cause of cancer-related mortality worldwide [1,2]. The poor prognosis associated with HCC is largely driven by its high recurrence rate and limited therapeutic window, which underscores the urgent need for early risk stratification to optimize treatment outcomes [1]. Vessels encapsulating tumor clusters (VETCs) are a specific vascular pattern characterized by tumor clusters being completely enveloped by CD34-positive endothelial cells [3]. Research has demonstrated that VETC positivity is an independent poor prognostic factor in HCC, associated with higher early recurrence rates and shorter disease-free and overall survival compared with that in patients with negative VETC [4]. Consequently, the noninvasive and accurate prediction of VETC status before treatment is crucial for identifying high-risk patients, tailoring personalized treatment strategies, and guiding postoperative surveillance [5]. Moreover, early identification of VETC status has the potential to influence clinical decisions regarding surgical versus nonsurgical management, thereby further improving treatment planning [6-8].

The traditional gold standard for VETC diagnosis relies on immunohistochemical analysis (eg, CD34 staining) of postoperative pathological specimens [9]. However, this method is invasive, cannot be performed preoperatively, and due to intratumoral heterogeneity, biopsy sampling may be subject to bias, potentially missing VETC-positive areas [10]. To overcome these limitations, noninvasive imaging techniques have been explored. Although conventional imaging techniques, such as computed tomography (CT) [11], magnetic resonance imaging (MRI), ultrasound [12], and 2-[¹⁸F]fluoro-2-deoxy-D-glucose positron emission tomography or computed tomography ([¹⁸F]FDG PET/CT) [13], are used for preoperative HCC evaluation and have been explored for predicting VETC status, their accuracy is limited by tumor heterogeneity, interobserver variability in interpretation, and the lack of standardized assessment criteria [14].

In recent years, artificial intelligence (AI) technologies based on medical images, particularly deep learning and radiomics, have shown significant potential for the noninvasive prediction of VETC and prognosis assessment in HCC [15]. Radiomics enables the high-throughput extraction of quantitative features from medical images, while deep learning models can automatically learn complex, subvisual imaging patterns associated with the VETC phenotype [16]. Unlike biopsy, which is limited by invasiveness and sampling bias, and unlike conventional imaging, which relies on subjective visual interpretation, AI-based approaches can comprehensively capture and integrate high-dimensional tumor characteristics to overcome these limitations [17]. However, existing studies exhibit considerable variability in model performance, suffer from small sample sizes, and lack methodological standardization, which collectively motivate the need for this systematic review and meta-analysis [18,19]. Moreover, the impact of different imaging modalities and algorithms on AI model performance remains controversial, lacking systematic synthesis [20].

Several systematic reviews have addressed related questions, but leave critical gaps that this study fills. Existing meta-analyses synthesizing radiomics models for HCC microvascular invasion have examined a different histopathological end point and therefore do not inform VETC-specific preoperative decision-making. A recent modality-specific meta-analysis by Wang et al [21] evaluated only MRI-based radiomics for VETC and did not pool evidence across CT, ultrasound, or PET/CT, nor did it quantify the prognostic value of AI-predicted VETC for early recurrence. Narrative reviews cataloging qualitative imaging features of VETC have not provided pooled diagnostic estimates, risk-of-bias appraisal using the recent Prediction model Risk Of Bias Assessment Tool (PROBAST)+AI tool, or GRADE (Grading of Recommendations, Assessment, Development, and Evaluation) certainty ratings [3]. To our knowledge, no previous quantitative synthesis has simultaneously (1) pooled diagnostic accuracy of AI-based VETC prediction across all 4 major contrast-enhanced modalities (contrast-enhanced magnetic resonance imaging [CEMRI], contrast-enhanced computed tomography [CECT], contrast-enhanced ultrasound [CEUS], and [^18^F]FDG PET/CT), (2) integrated PROBAST+AI risk-of-bias appraisal with GRADE certainty grading, and (3) meta-analyzed the hazard ratio (HR) of AI-predicted VETC for early recurrence. This review is designed to address these 3 gaps and therefore provides incremental, decision-relevant evidence beyond the existing literature.

Therefore, this systematic review and meta-analysis aims to synthesize the existing evidence to rigorously evaluate the overall diagnostic accuracy and sources of heterogeneity in image-based AI models for predicting VETC status and its prognostic value in HCC.

## Methods

### Overview

This systematic review and meta-analysis adhered to the PRISMA-DTA (Preferred Reporting Items for Systematic Reviews and Meta-Analyses for Diagnostic Test Accuracy) guidelines [22], CHARMS (Checklist for Critical Appraisal and Data Extraction for Systematic Reviews of Prediction Modelling Studies) checklists, PRISMA (Preferred Reporting Items for Systematic Reviews and Meta-Analyses) checklist, and PRISMA-S (Preferred Reporting Items for Systematic Reviews and Meta-Analyses literature search extension) checklist, and was prospectively registered on PROSPERO (CRD420251167155). These 4 checklists were provided in Checklists1^4^.

### Search Strategy

A comprehensive literature search was conducted across 4 major databases—PubMed (via NCBI), Embase (via Ovid), Web of Science (via Clarivate), and the Cochrane Library (via Wiley). The original search strategy was completed on July 11, 2025. During revision, the strategy was reconstructed to improve reproducibility and database-specific syntax; it was rerun on March 17, 2026, and the finalized strategy was last updated on April 17, 2026. Thus, July 11, 2025, refers to the initial search cutoff, whereas April 17, 2026, refers to the most recent rerun of the reconstructed search strategy. The reconstruction did not change the review question or eligibility criteria; it made the previously used AI-related terms, VETC-specific terms, and HCC-specific terms explicit for each database. To ensure comprehensiveness, no limits were placed on language or publication year. Search results from all databases were exported to EndNote X20 (Clarivate Analytics) for systematic deduplication, followed by manual verification. Moreover, 2 independent reviewers (XH and RF) performed initial screening of titles and abstracts, followed by full-text assessment.

### Inclusion and Exclusion Criteria

Studies were included based on the PITROS framework:

1. Population (P): patients with HCC scheduled for hepatectomy were included. Studies involving nonsurgical patients were excluded due to the lack of pathological confirmation, as definitive identification of the VETC vascular pattern currently requires microscopic evaluation of the resected tumor tissue.
2. Index test (I): radiomics-based AI, including CEMRI, CECT, CEUS, and [¹⁸F]FDG PET/CT, extracting high-dimensional quantitative features to predict VETC.
3. Target condition (T): positive group defined as VETC-positive, negative group defined as VETC-negative.
4. Reference standard (R): pathological biopsy of VETC for result validation.
5. Outcomes (O): sensitivity, specificity, area under the curve (AUC), and early recurrence HR.
6. Setting (S): retrospective or prospective case data from public databases or local hospitals.

Studies were systematically excluded based on the following comprehensive criteria: (1) titles and abstracts irrelevant to the review question; (2) nonoriginal literature types, including reviews, case reports, conference abstracts, meta-analyses, and editorial correspondence; (3) studies without AI algorithms; (4) research not evaluating VETC; (5) studies lacking essential diagnostic data; or (6) studies with overlapping patient data. For publications reporting more than 1 cohort, such as training, internal-validation, and external-validation cohorts, each cohort was treated as a separate dataset only when the patients were mutually exclusive. Cohorts with overlapping participants were not entered separately, in accordance with the principle that double counting occurs when the same participants or evidence are included more than once in a synthesis. The screening process was independently conducted by 2 reviewers (XH and RF), who first assessed title and abstract relevance, then evaluated full-text articles against inclusion and exclusion criteria. Any disagreements between reviewers were resolved through discussion, with a third reviewer (ZY) consulted if consensus could not be reached.

### Quality Assessment and GRADE Certainty

We used the latest PROBAST+AI quality assessment tool [23], which supersedes the 2019 PROBAST version. This comprehensive evaluation framework comprises 2 distinct phases—model development and model assessment, with each phase encompassing 7 critical domains addressing participants, data sources, predictive factors, outcome assessment, and analytical methodologies. The assessment categorizes each domain’s evaluation results into 3 levels—low (L), high (H), and unclear (U), determined through specific signaling questions. These questions were graded using a nuanced scale: “Yes” (Y), “Probably Yes” (PY), “Probably No” (PN), “No” (N), “No Information” (NI), and occasionally “Not Applicable” (NA).

To ensure methodological rigor and minimize potential bias, 2 independent reviewers (XH and RF) conducted a comprehensive risk of bias assessment using the PROBAST+AI tool. Any disagreements between reviewers were systematically resolved through in-depth discussion and meticulous analysis, ensuring high reliability and consistency in the final assessment. Detailed signaling questions and comprehensive evaluation tables are provided in Tables S2 and S3 in Multimedia Appendix 1.

We used the GRADE framework to assess the certainty of the evidence for sensitivity and specificity. This method evaluates 5 key domains—risk of bias, indirectness, inconsistency, imprecision, and small-study effects—to systematically rate the evidence quality. The detailed assessment criteria and protocols are fully documented in Table S4 in Multimedia Appendix 1 to ensure transparency.

### Outcome Measures

The primary outcome measures encompassed sensitivity, specificity, and AUC for AI-based approaches across different imaging modalities (CEMRI, CECT, CEUS, and [^18^F] FDG PET/CT), alongside their early recurrence HR values. Sensitivity, calculated as true positive (TP)/(TP+false negative [FN]), quantifies the AI model’s ability to accurately identify true positive cases, while specificity, calculated as true negative (TN)/(TN+false positive [FP]), evaluates the model’s proficiency in correctly recognizing negative cases. AUC represents the comprehensive discriminative performance of the receiver operating characteristic (ROC) curve, providing a holistic assessment of the model’s diagnostic capabilities. For early recurrence HR values, we directly extracted reported HRs with their 95% CIs from the original studies. In cases where direct reporting was absent, we planned to indirectly calculate HR values using published survival curves through precise data extraction and reconstruction techniques. An aggregated HR>1 with statistical significance would indicate the AI model’s significant prognostic stratification capability, demonstrating that patients categorized as “high-risk” by the AI model have a multiplicative early recurrence risk compared with the “low-risk” group. Early recurrence was defined according to the original studies; definitions varied across studies (eg,≤1 y or ≤2 y after surgery). For studies presenting multiple nonoverlapping patient datasets, we considered the contingency tables independent and extracted them comprehensively. When multiple AI algorithms were reported, we selected the highest-performing model based on AUC for analysis.

### Data Extraction

Two independent reviewers (XH and RF) systematically extracted data from full-text articles to assess their eligibility, with any discrepancies resolved through consultation with a third reviewer (ZY). The comprehensive data extraction process encompassed multiple critical domains, including (1) patient and study baseline information: authors, publication year, country, study design, imaging modalities (CEMRI, CECT, CEUS, and [^18^F] FDG PET/CT), reference standard, analysis methodology, total patient numbers (training and internal and external validation sets), and patient counts with positive VETC; (2) technical parameters: research center, data source, AI methodology, AI model, optimal AI algorithms, data splitting approach, and TP, FP, FN, and TN across different validation sets; and (3) imaging modality information: manufacturer, scanner modality, evaluation time, regions of interest, sequence parameters, enhancement phases, contrast agents, and radiotracer details. Given that most studies did not provide complete diagnostic contingency tables, we reconstructed TP, FP, FN, and TN values by systematically analyzing sensitivity, specificity, reference standard positive VETC cases, and total patient populations.

### Statistical Analysis

For the diagnostic meta-analysis, we addressed the observed between-study heterogeneity by using a bivariate random-effects model using the restricted maximum likelihood (REML) method. To enhance the robustness of our pooled estimates and account for uncertainty in variance, the Hartung-Knapp-Sidik-Jonkman (HKSJ) adjustment was applied to calculate the pooled sensitivity and specificity along with their corresponding 95% CIs. We calculated 95% prediction intervals (PIs) for the main diagnostic syntheses and for modality-specific subgroup syntheses only when random-effects pooling was performed with sufficient independent datasets. These PIs were reported as descriptive estimates of how performance might vary in a future clinical setting and were not used alone to justify additional subgroup analyses. Because subgroup analyses were prespecified by imaging modality, subgroup PIs were intended to contextualize heterogeneity within clinically defined modality groups rather than to generate new post hoc subgroup analyses [24]. The overall diagnostic accuracy was summarized by the AUC derived from the hierarchical summary receiver operating characteristic model, and the interpretation of these values was adjusted to acknowledge the impact of the substantial heterogeneity identified. Pooled HRs for early recurrence were estimated using a single prespecified REML+HKSJ random-effects model.

For CEMRI-based studies with more than 10 studies, meta-regression and bivariate boxplots were performed to investigate potential sources of heterogeneity. The prespecified covariates for meta-regression included center (single vs multi), validation type (internal vs external), data splitting method (hold-out vs independent validation), AI model type (radiomics vs radiomics and clinical), AI algorithms (deep learning vs machine learning), and MRI field strength (1.5T&3.0T vs 3.0T). The impact of different algorithms on diagnostic performance was also assessed. A bubble plot visualized the diagnostic odds ratio (DOR) over time. The clinical utility of CEMRI-based models was appraised using Fagan’s nomogram, and small-study effects were assessed using Deeks’ funnel plot asymmetry test (*P* value<.05 for the slope coefficient, indicating potential bias). A chord diagram illustrated the frequency of different imaging modalities and algorithms. All analyses were conducted using Stata (version 15.1; StataCorp LLC; with the *Midas* and *Metadta* packages) and R (version 4.3.1; R Core Team; using ggplot2 and *tidyverse*). Several deviations from the original registered protocol should be noted; the HKSJ adjustment and the reporting of 95% PIs were not prespecified but were incorporated post hoc to strengthen methodological rigor. Additionally, certain meta-regression covariates, such as MRI field strength and AI algorithm type, were added based on clinical relevance identified during the review process. No changes were made to the original inclusion or exclusion criteria.

## Results

### Study Selection

**Figure 1.**
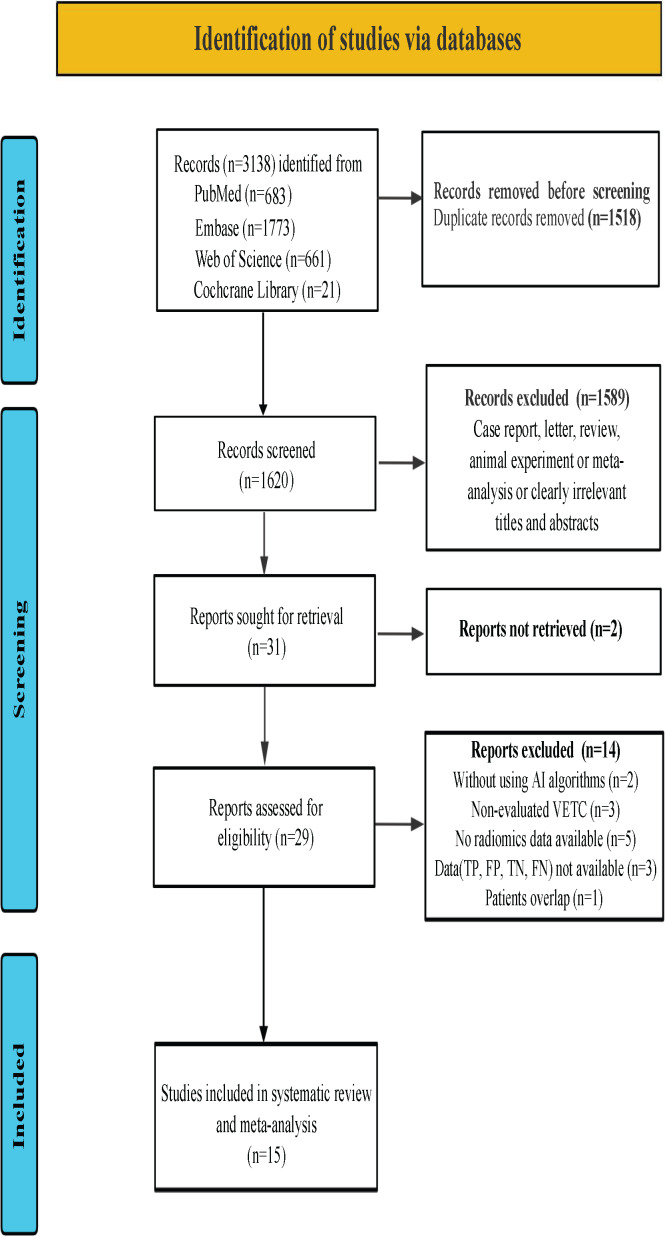
PRISMA (Preferred Reporting Items for Systematic Reviews and Meta-Analyses) flow diagram illustrating the systematic identification and selection process for studies on radiomics-based AI models predicting vessels encapsulating tumor clusters in hepatocellular carcinoma. AI: artificial intelligence; FN: false negative; FP: false positive; TN: true negative; TP: true positive; VETC: vessels encapsulating tumor cluster.

The initial database search identified 549 potentially relevant publications. Following the removal of 281 duplicate records, 268 articles proceeded to the preliminary screening phase. During this stage, 243 records were excluded based on title and abstract review, primarily due to clear irrelevance or nonconforming publication types. This resulted in 25 articles sought for full-text retrieval. Out of 25 articles, 1 was excluded as the full text was inaccessible. The remaining 24 articles underwent a detailed, full-text eligibility assessment. Of these, 9 studies were excluded due to insufficient or incomplete diagnostic data, with reasons including (1) without using AI algorithms, (2) nonevaluated VETC, (3) data (TP, FP, TN, and FN) not available, or (4) patients overlap. Consequently, 15 studies [6-8,12,24-34] met the predefined inclusion criteria and were incorporated into the final meta-analysis. The entire study selection process adhered to the PRISMA guidelines and is detailed in Figure 1.

### Study Description and Quality Assessment

A total of 15 eligible studies were included in this meta-analysis (Tables1  2). The validation cohorts consisted of 12 studies [6-8,12,26-28,31-35] providing internal validation data (encompassing 729 patients) and 7 studies [6,7,27,29,30,32,36] providing external validation data (encompassing 613 patients). The distribution by primary imaging modality was as follows: 3 used CECT [6,27,36], 1 used [^18^F]FDG PET/CT [8], 1 used CEUS [12], and the remaining 10 studies used CEMRI [7,26,28-35]. All 15 studies, published between 2021 and 2025, were retrospective in design. The reference standard for confirming VETC status was pathological biopsy. With the exception of 1 study from Japan [35], all others originated from China. All analyses were conducted on a per-patient basis. When a publication reported training, internal-validation, and/or external-validation cohorts, these cohorts were extracted as separate datasets only if the original article indicated that the patients did not overlap.

**Table 1.** Study and patient characteristics of the included studies.

Author	Year	Country	Study design	Type of imaging	Temporal range	Reference standard	Analysis	Total patients or lesions, n			No. of VETC^a^ patients or lesions
								Training	Internal validation	External validation	
Zhao et al [6]	2025	China	Retro^b^	CECT^c^	2014‐2022	Pathology	PB^d^	169	72	152	Training: 39 Internal validation: 14 External validation: 53
Xu et al [12]	2024	China	Retro	CEUS^e^	2018‐2021	Pathology	PB	195	47	—^f^	Training: 80 Internal validation: 16
Matsuda et al [33]	2025	Japan	Retro	CEMRI	2008‐2020	Pathology	PB	153	51	—	Training: 33 Internal validation: 12
Hu et al [8]	2025	China	Retro	PET/CT^g^	2015‐2022	Pathology	PB	103	46	—	Training: 55 Internal validation: 23
Gu et al [30]	2025	China	Retro	CEMRI	2015‐2022	Pathology	PB	144	54	32	Training: 73 Internal validation: 28 External validation: 16
Che et al [7]	2025	China	Retro	CEMRI	2014‐2021	Pathology	PB	253	108	144	Training: 82 Internal validation: 34 External validation: 54
Zhang et al [24]	2024	China	Retro	CECT	2017‐2023	Pathology	PB	106	47	37	Training: 53 Internal validation: 23 External validation: 18
Dong et al [31]	2023	China	Retro	DCE-MRI^i^	2015‐2021	Pathology	PB	154	67	—	Training: 46 Internal validation: 22
Zhang et al [25]	2024	China	Retro	CEMRI^h^	2013‐2020	Pathology	PB	163	71	—	Training: 71 Internal validation: 30
Yang et al [27]	2024	China	Retro	DCE-MRI	2015‐2021	Pathology	PB	219	—	101	Training: 68 External validation: 25
Qu et al [29]	2023	China	Retro	CEMRI	2014‐2021	Pathology	PB	168	72	—	Training: 74 Internal validation: 31
Chu et al [32]	2022	China	Retro	CEMRI	2017‐2020	Pathology	PB	93	40	—	Training: 35 Internal validation: 16
Yu et al [26]	2021	China	Retro	CEMRI	2015‐2020	Pathology	PB	128	54	—	Training: 72 Internal validation: 25
Wang et al [28]	2025	China	Retro	CEMRI	2016‐2023	Pathology	PB	305	—	115	Training: 161 External validation: 55
Wang et al [34]	2025	China	Retro	CECT	2017‐2022	Pathology	PB	130	—	32	Training: 68 External validation: 14

a VETC: vessels encapsulating tumor cluster.

b Retro retrospective.

c CECT: contrast-enhanced computed tomography.

d PB: patient-based.

e CEUS: contrast-enhanced ultrasonography.

f Not available.

g PET/CT: positron emission tomography/computed tomography.

h CEMRI: contrast-enhanced magnetic resonance imaging.

i DCE-MRI: dynamic contrast-enhanced magnetic resonance imaging.

**Table 2.** Technical aspects of included studies.

Author	Year	Center	Data source	AI^a^ method	AI model	Optimal AI algorithms^b^	Data splitting method	Internal validation sets				External validation sets			
								TP^c^	FP^d^	FN^e^	TN^f^	TP	FP	FN	TN
Zhao et al [6]	2025	Multiple centers	Local hospital	ML^g^	R&CM^h^	LR^i^	Hold-out	12	17	2	41	40	21	13	78
Xu et al [12]	2024	Single center	Local hospital	DL^j^	RM^k^	CNN^l^	Hold-out	13	6	3	25	—^m^	—	—	—
Matsuda et al [33]	2025	Single center	Local hospital	ML	R&CM	LASSO^n^	Hold-out	10	9	2	30	—	—	—	—
Hu et al [8]	2025	Multiple centers	Local hospital	ML	R&CM	LR	Independent validation	—	—	—	—	17	6	6	17
Gu et al [30]	2025	Multiple centers	Local hospital	DL	R&CM	MLP^o^	Independent validation	—	—	—	—	24	4	4	22
Gu et al [30]	2025	Multiple centers	Local hospital	DL	R&CM	MLP	Independent validation	—	—	—	—	15	2	1	14
Che et al [7]	2025	Multiple centers	Local hospital	ML	R&CM	LR	Hold-out	26	21	8	53	43	27	11	63
Zhang et al [24]	2024	Multiple centers	Local hospital	ML	R&CM	LR	Hold-out	19	6	4	18	11	3	7	16
Dong et al [31]	2023	Single center	Local hospital	DL	RM	DNN^p^	Time-series split	17	8	5	37	—	—	—	—
Zhang et al [25]	2024	Single center	Local hospital	ML	R&CM	LR	Hold-out	30	12	0	29	—	—	—	—
Yang et al [27]	2024	Multiple centers	Local hospital	DL	RM	CNN	Independent validation	—	—	—	—	20	30	5	46
Qu et al [29]	2023	Single center	Local hospital	ML	R&CM	LR	Hold-out	27	15	4	26	—	—	—	—
Chu et al [32]	2022	Single center	Local hospital	DL	R&CM	CNN	Hold-out	7	0	9	24	—	—	—	—
Yu et al [26]	2021	Single center	Local hospital	ML	R&CM	RF^q^	Hold-out	25	4	0	25	—	—	—	—
Wang et al [28]	2025	Multiple centers	Local hospital	DL	R&CM	CNN	Independent validation	—	—	—	—	37	8	18	52
Wang et al [34]	2025	Single center	Local hospital	ML	RM	RF	Hold-out	—	—	—	—	8	3	6	15

a AI: artificial intelligence.

b The optimal artificial intelligence algorithm means the algorithm with the highest area under the curve value.

c TP: true positive.

d FP: false positive.

e FN: false negative.

f TN: true negative.

g ML: machine learning.

h R&CM: radiomic and clinical model.

i LR: logistic regression.

j DL: deep learning.

k RM: radiomic model.

l CNN: convolutional neural network.

m Not available.

n LASSO: least absolute shrinkage and selection operator.

o MLP: multilayer perceptron.

p DNN: deep neural network.

q RF: random forest.

**Figure 2.**
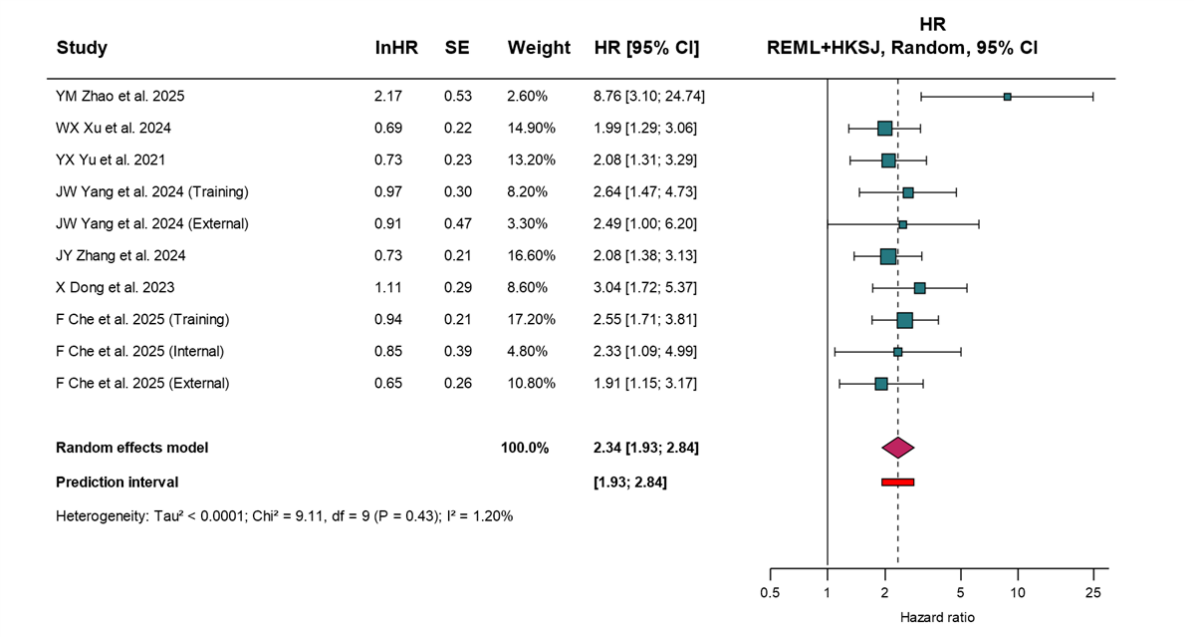
Rescaled forest plot of hazard ratios for early recurrence risk (early recurrence as defined by the original studies, eg, ≤1 or ≤2 years post hepatectomy or transplantation) in patients with hepatocellular carcinoma stratified by artificial intelligence–predicted vessels encapsulating tumor cluster status across multiple imaging modalities. The x-axis is displayed on a logarithmic scale with an adjusted range to improve visualization of CIs and the prediction interval. Rows from the same article indicate mutually exclusive training, internal-validation, or external-validation cohorts when reported. HKSJ: Hartung-Knapp-Sidik-Jonkman; HR: hazard ratio; REML: restricted maximum likelihood [6,7,12,25-27,31].

The methodological quality of the included studies was appraised using the PROBAST+AI tool, with the detailed signaling-question tables provided in Tables S2 and S3 in Multimedia Appendix 1. For model development, the overall quality judgment was high-risk in 6.7% (1/15) of studies and low-risk in 93.3% (14/15). Applicability concerns for model development were high in 13.3% (2/15) of studies and low in 86.7% (13/15). For model validation, the overall risk of bias was high in 20% (3/15) of studies, unclear in 20% (3/15), and low in 60% (9/15). Applicability concerns for validation cohorts were low in all included studies. The main high-risk judgments were driven by analysis-related issues, whereas unclear judgments mainly reflected insufficient reporting of outcome assessment. These risk-of-bias findings are presented in the main manuscript together with the PRISMA flow diagram (Figure 1) and the prognostic forest plot (Figure 2), with full details retained in Multimedia Appendix 1.

The certainty of evidence was evaluated according to the GRADE framework and is now summarized in a main-text diagnostic Summary of Findings table. The table presents expected TP, FN, TN, and FP results per 1000 patients tested at the median VETC prevalence of 38%, together with the number of participants and studies, certainty ratings, and concise comments. Detailed domain judgments remain in Table S4 in Multimedia Appendix 1. Across outcomes, certainty ranged from low to moderate, with downgrading mainly driven by validation-related risk of bias, imprecision, and heterogeneity.

### The Predictive Performance and Temporal Development Trends of Different AI Algorithms

**Figure 3.**
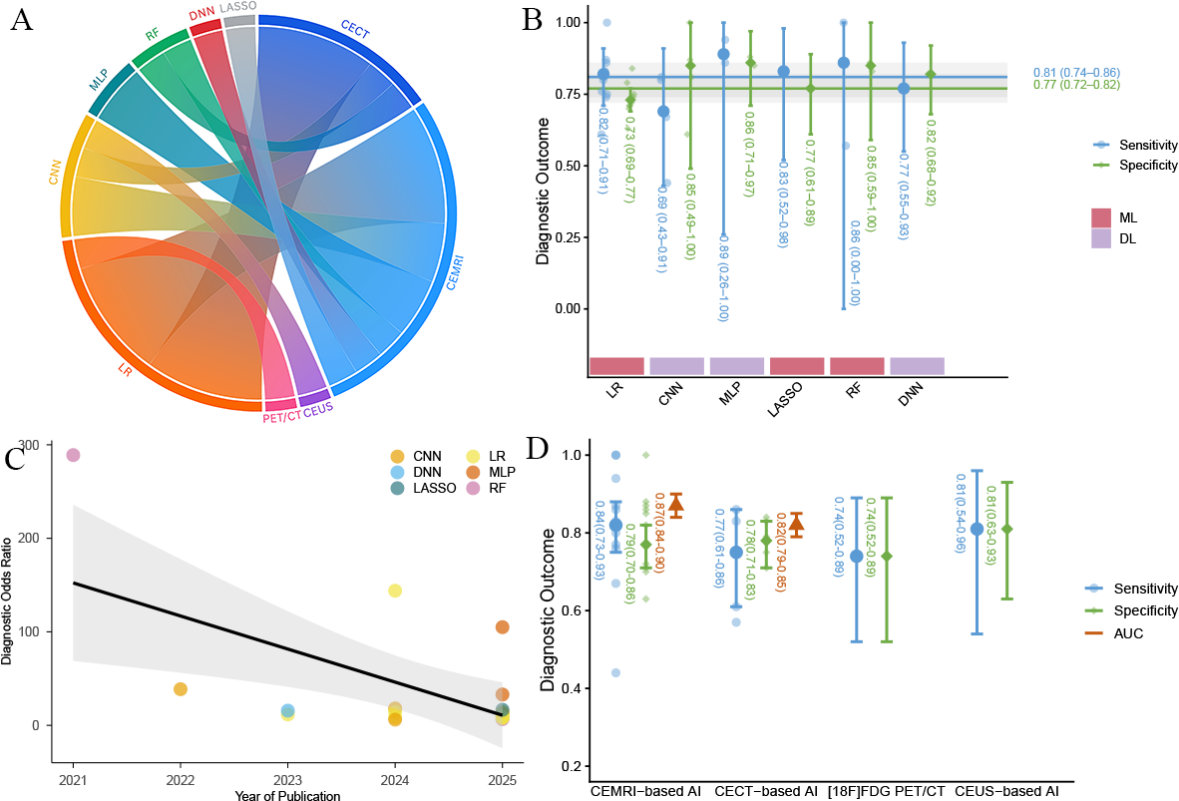
Comprehensive visualization of the interactions between imaging modalities and artificial intelligence (AI) algorithms, and the diagnostic performance of AI models for predicting vessels encapsulating tumor clusters in hepatocellular carcinoma. It includes: (A) a chord diagram illustrating the interconnections between 4 imaging modalities (contrast-enhanced magnetic resonance imaging, contrast-enhanced computed tomography, contrast-enhanced ultrasound, and 2-[¹⁸F]fluoro-2-deoxy-D-glucose positron emission tomography or computed tomography) and 9 AI algorithms; (B) violin plots comparing the predictive performance (area under the curve) of different AI algorithms; (C) a bubble plot showing the diagnostic odds ratio of the optimal-performing AI algorithm stratified by publication year; and (D) violin plots summarizing the diagnostic performance (sensitivity and specificity) of various imaging modalities. AI: artificial intelligence; AUC: area under the curve; CECT: contrast-enhanced computed tomography; CEMRI: contrast-enhanced magnetic resonance imaging; CEUS: contrast-enhanced ultrasound; CNN: convolutional neural network; DNN: deep neural network; LASSO: least absolute shrinkage and selection operator; LR: logistic regression; MLP: multilayer perceptron; RF: random forest.

As illustrated in Figure 3B, the multilayer perceptron (MLP) algorithm demonstrated the highest sensitivity and specificity values at 0.89 (95% CI 0.26‐1.00) and 0.86 (95% CI 0.71‐0.97). Furthermore, Figure 3C indicates a slight decline in the DOR from 2021 to 2025, with random forest (RF) achieving relatively high DOR values while other algorithms maintained lower levels.

### The Predictive Performance of Different Imaging-Based AI Model

The results indicated that no significant threshold effect was observed for AI models based on CEMRI (Spearman ρ=−0.03, *P*=.93). The primary pooled analysis combined both internal and external validation datasets. For CEMRI-based AI models, the average pooled sensitivity was 0.84 (95% CI 0.73‐0.93; 95% PI 0.45‐1.00; τ^2^=0.03) with low certainty, and the average pooled specificity was 0.79 (95% CI 0.70‐0.86; 95% PI 0.50‐0.97; τ²=0.02) with low certainty. The 95% CIs describe uncertainty around the average pooled estimates, whereas the 95% PIs describe how accuracy may be distributed in a future setting; therefore, the wide CEMRI PIs indicate clinically important between-setting variability despite favorable average accuracy. For CECT-based AI models, sensitivity was 0.75 (95% CI 0.61‐0.86; 95% PI 0.62‐0.85; τ²<0.001) with moderate certainty, and specificity was 0.78 (95% CI 0.71‐0.83; 95% PI 0.69‐0.85; τ²=0) with moderate certainty. For [^18^F]FDG PET/CT and CEUS, only 1 study was available for each modality; therefore, their average estimates were not used to infer a distribution of effects across settings. The [^18^F]FDG PET/CT-based AI model had sensitivity and specificity of 0.74 (95% CI 0.52‐0.90), both with low certainty. The CEUS-based AI model had sensitivity of 0.81 (95% CI 0.54‐0.96) and specificity of 0.81 (95% CI 0.63‐0.93), both with low certainty.

### The Predictive Value of Early Recurrence

The GRADE summary of findings, including the diagnostic summary of findings estimates and expected results per 1000 patients tested, is presented in Table 3.

As illustrated in Figure 2, regarding the prognostic value for predicting early recurrence, the pooled analysis of 10 nonoverlapping datasets from 7 studies [6,7,12,25-27,31] revealed an average HR of 2.34 (95% CI 1.93‐2.84) with the REML+HKSJ method. The CI reflects uncertainty around the average prognostic effect. Because between-study heterogeneity was very low (τ²<0.0001), the PI was similar to the CI, suggesting limited observed dispersion for the pooled HR; however, modality-specific HRs from single studies should not be interpreted as distributions across settings. In this survival-HR forest plot, Che et al [7] contributed 3 mutually exclusive patient cohorts (training, internal validation, and external validation), and Yang et al [27] contributed 2 mutually exclusive cohorts (training and external validation). Because these patient groups did not overlap, they were treated as separate datasets and did not constitute double-counting. When stratified by imaging modality, the HR was 2.32 (95% CI 2.04‐2.63; τ²=0) for CEMRI-based AI (8 datasets from 5 studies [7,25-27,31]), 8.76 (95% CI 3.10‐24.74) for CECT-based AI (1 study [6]), and 1.99 (95% CI 1.29‐3.06) for CEUS-based AI (1 study [12]). The extremely high HR observed in the CECT subgroup should be interpreted cautiously, as it was derived from only a single study with a wide CI. Similarly, the CEUS-based estimate was based on a single study.

**Table 3.** GRADE^a^ summary of findings table for AI^b^-based imaging prediction of VETC^c^ in HCC^d^. Expected results were calculated per 1000 patients tested at the median VETC prevalence of 38% and rounded to the nearest whole patient. Certainty was judged using the GRADE framework for diagnostic test accuracy evidence; downgrading reflected validation-related risk of bias, imprecision, heterogeneity or inconsistency, limited external validation, and small-study or single-study evidence where applicable. Prediction intervals were not estimable for modalities represented by a single study.

Insex test and test result	Number of results per 1000 patients tested (95% CI), at 38% VETC prevalence	Participants and studies or datasets	Certainty of the evidence (GRADE)	Comments
CEMRI^e^-based AI^b^		909 participants (10 studies or datasets)	Low	Favorable average accuracy, but certainty was downgraded for validation-related risk of bias, imprecision, and heterogeneity/inconsistency; prediction intervals were wide.
True positives	319 (277-353)			
False negatives	61 (27-103)			
True negatives	490 (434-533)			
False positives	130 (87-186)			
CECT^f^-based AI		340 participants (3 studies or datasets)	Moderate	Average accuracy was moderate-to-good; certainty was mainly limited by imprecision and limited external validation.
True positives	285 (232-327)			
False negatives	95 (53-148)			
True negatives	484 (440-515)			
False positives	136 (105-180)			
[^18^F]FDG PET/CT^g^-based AI		46 participants (1 study or dataset)	Low	Single-study evidence was preliminary; certainty was downgraded for imprecision and limited validation, and prediction intervals were not estimable.
True positives	281 (198-342)			
False negatives	99 (38-182)			
True negatives	459 (322-558)			
False positives	161 (62-298)			
CEUS^h^-based AI		47 participants (1 study or dataset)	Low	Single-study evidence suggested potential accuracy but requires independent validation; prediction intervals were not estimable.
True positives	308 (205-365)			
False negatives	72 (15-175)			
True negatives	502 (391-577)			
False positives	118 (43-229)			

a GRADE: Grading of Recommendations, Assessment, Development, and Evaluation.

b AI: artificial intelligence.

c VETC: vessels encapsulating tumor cluster.

d HCC: hepatocellular carcinoma.

e CEMRI: contrast-enhanced magnetic resonance imaging.

f CECT: contrast-enhanced computed tomography.

g [18F]FDG PET/CT: fluorine-18 fluorodeoxyglucose positron emission tomography/computed tomography.

h CEUS: contrast-enhanced ultrasonography.

### Meta-Regression and Bivariate Box Plots

**Figure 4.**
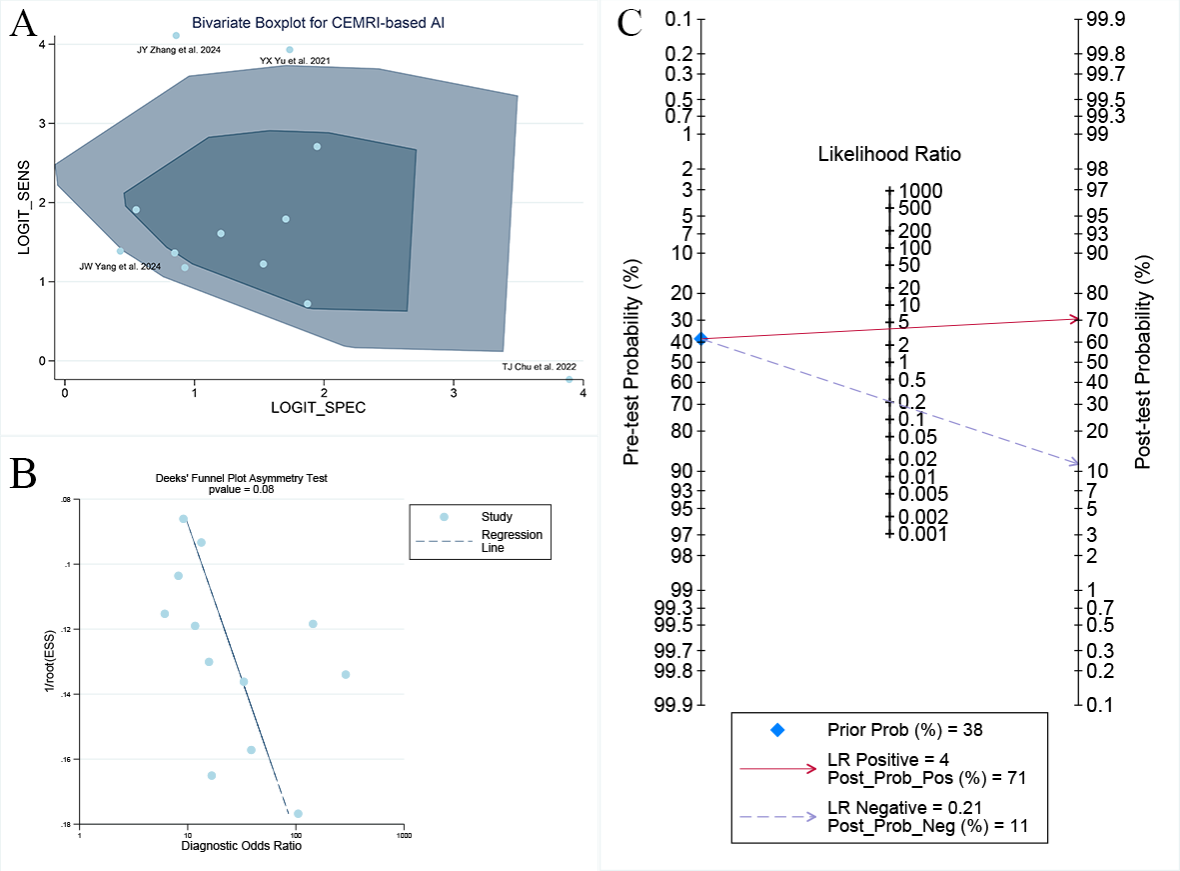
Heterogeneity evaluation, small effect test, and clinical utility analysis of artificial intelligence models for predicting vessels encapsulating tumor clusters in hepatocellular carcinoma. (A) Bivariate boxplot of sensitivity and specificity, visualizing the distribution and heterogeneity of study-level estimates. (B) Deek’s funnel plot for the assessment of potential small-study effects. (C) Fagan plot for Bayesian analysis of clinical utility, using the median vessels encapsulating tumor cluster prevalence (38%) across included studies as the prior probability. AI: artificial intelligence; CEMRI: contrast-enhanced magnetic resonance imaging; LR: logistic regression [25-27,32].

The results of the meta-regression are illustrated in Table 4 and indicate that the heterogeneity in CEMRI-based AI models was primarily attributable to variations in center (specificity: *P*=.01); validation (specificity: *P*=.01); AI algorithms (specificity: *P*<.001); MRI field strength (specificity: *P*=.05). Specificity was significantly higher in single-center studies compared with multicenter studies (0.81 vs 0.75, *P*=.01). Similarly, internal validation cohorts demonstrated significantly higher specificity than external validation cohorts (0.81 vs 0.75, *P*=.01). Deep learning models achieved significantly higher specificity than machine learning models (0.83 vs 0.73, *P*<.001). Furthermore, studies using a 3.0T MRI field strength showed significantly higher specificity than those using a combination of 1.5T and 3.0T scanners (0.79 vs 0.77, *P*=.05).

it The bivariate box plot analysis identified the studies by Yu et al [26], Zhang et al [25], Yang et al [27], and Chu et al [32] as outliers, suggesting they may be potential sources of heterogeneity (Figure 4A).

**Table 4.** Subgroup analysis and meta-regression analysis of CEMRI^a^-based AI^b^ performance.

Subgroup	Studies, n	Sensitivity (95%CI)	Meta-regression *P* value	Specificity (95%CI)	Meta-regression *P* value
Center			.06		.01
Single center	6	0.87 (0.77‐0.96)		0.81 (0.72‐0.90)	
Multicenter	6	0.81 (0.69‐0.92)		0.75 (0.65‐0.85)	
Validation			.06		.01
Internal validation	6	0.87 (0.77‐0.96)		0.81 (0.72‐0.90)	
External validation	6	0.81 (0.69‐0.92)		0.75 (0.65‐0.85)	
Data splitting			.31		.18
Hold out splitting	7	0.86(0.75,0.96)		0.77 (0.68‐0.86)	
Independent validation	4	0.83 (0.68‐0.98)		0.79 (0.67‐0.91)	
AI^b^ model			.95		.56
Radiomic	2	0.80 (0.57‐1.00)		0.71 (0.55‐0.88)	
Radiomic and clinical	10	0.85 (0.76‐0.93)		0.79 (0.72‐0.86)	
AI algorithms			.98		＜.001
Deep learning	6	0.77 (0.64‐0.89)		0.83 (0.75‐0.91)	
Machine learning	6	0.89 (0.82‐0.97)		0.73 (0.64‐0.82)	
MRI^c^ field strength			.37		.05
1.5 T and 3.0 T	5	0.85 (0.73‐0.97)		0.77 (0.67‐0.88)	
3.0 T	7	0.83 (0.72‐0.94)		0.79 (0.70‐0.87)	

a CEMRI: contrast-enhanced magnetic resonance imaging.

b AI: artificial intelligence.

c MRI: magnetic resonance imaging.

### Sensitivity Analysis

Sensitivity analysis was performed by excluding the 4 outlier studies [25-27,32] to assess their impact on the pooled estimates for CEMRI-based AI models. After exclusion, the pooled sensitivity was 0.80 (95% CI 0.73‐0.87), specificity was 0.77 (95% CI 0.70‐0.84), and AUC was 0.85 (95% CI 0.82‐0.88), showing no substantial changes compared with the primary results. Additionally, a subgroup analysis was conducted by including only pure radiomics studies. In this analysis, the pooled sensitivity, specificity, and AUC for CEMRI-based models were 0.81 (95% CI 0.68‐0.92), 0.68 (95% CI 0.50‐0.84), and 0.75 (95% CI 0.71‐0.79), indicating a slight decrease in diagnostic performance. We also performed an analysis restricted to studies with external validation. In this analysis, the pooled sensitivity, specificity, and AUC of CEMRI-based models were 0.80 (95% CI 0.67‐0.90), 0.77 (95% CI 0.61‐0.90), and 0.84 (95% CI 0.80‐0.87), respectively.

### Small-Study Effects and Clinical Application Value

Deeks’ funnel plot asymmetry test indicated no statistically significant small-study effects for CEMRI-based AI models (*P*=.08; Figure 4B). Furthermore, using the prespecified median prevalence of 38% as the pretest probability, the Fagan nomogram demonstrated a positive posttest probability of 71% and a negative posttest probability of 11% (Figure 4C).

## Discussion

### Main Findings

Our meta-analysis demonstrated that AI models based on CEMRI yielded the highest sensitivity (0.84) and AUC (0.87) for predicting VETC in HCC, while models based on CEUS achieved the highest specificity (0.81). The superior sensitivity and AUC of CEMRI-based AI can be attributed to the ability of CEMRI to provide high soft-tissue contrast and detailed visualization of intratumoral heterogeneity and peritumoral vasculature [26]. This allows AI models to extract a large number of high-dimensional radiomic features, potentially maximizing the capture of subtle image phenotypes associated with the VETC pattern [35]. Conversely, the high specificity of CEUS-based AI likely stems from its capacity for real-time assessment of tumor hemodynamics and microvascular perfusion [36]. AI models can leverage this dynamic information to accurately identify the characteristic perfusion patterns of VETC-positive HCCs, thereby reducing false-positive diagnoses [37]. Furthermore, the typical enhancement patterns of non-VETC HCCs on CEUS may allow AI models to more reliably exclude VETC-negative cases [12]. However, as this finding was based on a single study, it should be interpreted with caution and requires validation in larger, independent cohorts.

### Detailed Discussion and Comparison

Regarding the impact of different algorithms, our subgroup analysis revealed that the RF algorithm demonstrated the highest sensitivity (1.00), while both MLP and RF achieved the highest specificity values (0.86). The robust performance of RF, characterized by high sensitivity and specificity, can be explained by its ensemble nature. By constructing a multitude of decision trees and aggregating their predictions, RF effectively mitigates overfitting, often seen in single models, thereby enhancing its ability to correctly identify both positive and negative cases [38]. Furthermore, RF’s inherent capability to automatically assess feature importance allows it to select the most discriminative radiomics features for classification, improving its adaptability and discriminatory power when dealing with complex, high-dimensional medical imaging data [39]. However, the perfect sensitivity of 1.00 reported for the RF model should be interpreted with caution, as this finding may reflect study-specific characteristics, such as small sample sizes, overfitting, or the use of internal validation only. The high specificity attained by MLP, an artificial neural network, likely stems from its capacity to model complex, nonlinear relationships within the data. When the feature distribution of negative (non-VETC) samples is distinct, MLP can leverage its multiple hidden layers to learn highly specific decision boundaries, effectively separating them from positive cases and resulting in a low false-positive rate [40]. However, conclusions regarding MLP performance remain preliminary due to the limited number of studies.

Our pooled prognostic analysis, based on 10 nonoverlapping datasets from 7 studies [6,7,12,25-27,31], indicated that patients with HCC classified as VETC-positive by AI models had a significantly higher risk of early recurrence, with an HR of 2.34 (95% CI 1.93‐2.84). This suggests that AI-based VETC prediction offers prognostic capability for early recurrence risk. Notably, the AI model based on CECT yielded the highest HR of 8.76, although its wide 95% CI (3.10‐24.74) warrants caution in interpretation, likely attributable to the limited number of studies available for this modality. Given the small number of contributing studies, this estimate may be unstable. Previous research findings indicated that in the pathological classification, the VETC-positive group had an HR (95% CI 1.44‐2.07) for early recurrence [21], and the results of this study suggest that the AI model’s predictive capability for early recurrence risk is not inferior to that of traditional pathological risk classification.

A recent meta-analysis by Wang et al [41] specifically evaluated the performance of MRI-based radiomics for predicting VETC in HCC, reporting a sensitivity of 0.88, specificity of 0.86, and an AUC of 0.93. This meta-analysis, which included a broader scope of imaging modalities, found a comparatively lower pooled predictive performance for CEMRI-based AI. This discrepancy may be explained by our inclusion of a larger number of studies investigating MRI-based AI models, which provides a more generalizable, albeit slightly less optimistic, estimate of real-world performance. Our study extends the findings of Wang et al [41] by providing a comprehensive comparative analysis of AI performance across multiple imaging modalities, including CEUS, CECT, and [^18^F] FDG PET/CT, in addition to CEMRI. We further investigated the influence of different AI algorithms on predictive performance. Most significantly, our work provides a novel contribution by quantitatively synthesizing the prognostic value of AI-predicted VETC status, demonstrating that the AI model exhibits good prognostic capability for predicting early recurrence.

Significant heterogeneity was observed among the included studies, which is an acknowledged challenge in radiomics meta-analyses. We accounted for this inherent variability a priori by using a bivariate random-effects hierarchical summary ROC model with HKSJ adjustment for diagnostic metrics, and a prespecified REML+HKSJ random-effects model for the prognostic HR synthesis, with DerSimonian-Laird and uncorrected REML reported only as sensitivity analyses. Meta-regression and bivariate boxplot analysis identified several potential sources of this heterogeneity. The significantly higher specificity in single-center versus multicenter studies (0.81 vs 0.75, *P*=.01) may reflect the greater standardization in imaging protocols and patient populations within a single institution, whereas multicenter studies encompass variations in scanners, acquisition parameters, and patient demographics that can challenge model generalizability [42]. The higher specificity in internal validation cohorts compared with external validation sets (0.81 vs 0.75, *P*=.01) underscores the performance drop commonly encountered when models are applied to independent, external data with different distributions [43]. The superior specificity of deep learning models over machine learning models (0.83 vs 0.73, *P*<.001) may be attributed to their ability to automatically learn complex, hierarchical feature representations directly from images, potentially capturing more discriminative patterns to rule out negative cases [44]. Furthermore, studies using 3.0T MRI scanners showed higher specificity than those using a mix of 1.5T/3.0T devices (0.79 vs 0.77, *P*=.05), likely due to the superior signal-to-noise ratio and spatial resolution of 3.0T, leading to more consistent and detailed image data [45]. The bivariate boxplot also pinpointed specific studies as potential outliers contributing to heterogeneity [24,27,28,33].

### Future Direction

The findings of this meta-analysis hold several implications for clinical practice. The identified strengths of specific imaging-modality and algorithm combinations—such as the high sensitivity of CEMRI-based AI and the high specificity of CEUS-based AI—could inform the development of noninvasive tools for preoperative VETC prediction and risk stratification [46]. The demonstrated prognostic value of AI-classified VETC status for early recurrence, which was comparable with pathological classification, suggests its potential role in guiding postoperative surveillance and adjuvant therapy decisions, thereby facilitating timely management of HCC [47]. It is crucial to emphasize that any implemented AI should function as a decision-support tool, augmenting rather than replacing clinician judgment [48]. A key consideration for clinical implementation is model generalizability; the fact that only 7 included studies [6,7,24,27,28,30,34] reported external validation performance underscores the need for future research to prioritize robust, multicenter external validation [42]. Current unimodal AI models also have inherent limitations, as they may not capture the comprehensive information available from a multiparametric imaging workup [43]. Future AI systems may need to integrate findings across multiple imaging modalities, correlate them with clinical context, and communicate synthesized insights [44]. Other barriers to widespread adoption include the scarcity of expert-annotated data, regulatory hurdles, and challenges regarding model interpretability and transparency [45]. The “black-box” nature of many complex models remains a significant obstacle to clinical trust and integration [49]. Advances in techniques, like few-shot learning, self-supervised models, and explainable AI, alongside collaborative platforms, are needed to build a robust and trustworthy AI ecosystem in radiology [50]. AI-assisted VETC prediction may be particularly beneficial for high-risk surgical candidates, such as patients with large tumors, borderline resectable disease, or elevated AFP levels [51].

### Limitations

Several limitations of this meta-analysis should be acknowledged when interpreting the results. First, the predominantly retrospective design and relatively small sample sizes of the included studies may introduce potential biases in patient selection and data collection. Therefore, large-scale, prospectively designed studies are warranted to validate these findings [52]. Second, to avoid potential patient overlap across multiple algorithms reported in a single study, we extracted data only for the best-performing algorithm from each publication. While this approach mitigates bias, it prevents a comprehensive evaluation of the performance spectrum across all developed algorithms and may present an overly optimistic view; future studies with independent test sets for all models are needed [53]. Third, the generalizability of our conclusions may be limited by the geographical distribution of the included literature. Specifically, 14 out of the 15 included studies (93.3%) were conducted in China, representing a significant geographic concentration within Asian populations. While these findings are robust for the studied cohorts, validation in more ethnically and geographically diverse populations outside of China is necessary to ensure the widespread applicability and diagnostic stability of these AI models across different health care systems and genetic backgrounds [54]. Fourth, the average pooled diagnostic effects should be distinguished from the distribution of effects across settings. The 95% CIs describe uncertainty around the average sensitivity or specificity, whereas the 95% PIs describe how performance may vary in future settings. For example, the CEMRI sensitivity CI was 0.73‐0.93, but the PI was 0.45‐1.00, indicating that substantial heterogeneity may remain despite favorable average performance, as recommended by Borenstein [55] for the interpretation of heterogeneity. Fifth, the GRADE certainty of evidence was low for most outcomes and moderate only for CECT sensitivity and specificity, mainly because of imprecision, validation-related risk of bias, and limited external validation. Given these limitations, the pooled results should be interpreted cautiously to avoid overstatement. Future studies with standardized imaging protocols, unified reporting guidelines, prospective designs, and geographically diverse external validation are needed to reduce heterogeneity and improve certainty. Finally, differences in the definition of early recurrence across studies may have influenced the pooled HR estimates [56], and other potential sources of heterogeneity—such as variations in patient demographics, tumor stage, and imaging acquisition protocols—may also contribute to the observed variability.

### Conclusion

In conclusion, the available evidence indicates that AI models derived from contrast-enhanced imaging can identify VETC status with clinically meaningful average accuracy, particularly for CEMRI, and that model-predicted VETC positivity is associated with a higher risk of early recurrence. The main contribution of this synthesis is to define the current evidentiary boundary for preoperative VETC risk stratification; AI outputs may help clinicians recognize tumors with aggressive vascular architecture, select patients for intensified follow-up, and generate hypotheses for individualized perioperative planning. These applications remain conditional. Most included cohorts were retrospective and geographically concentrated, external validation was sparse, and uncertainty was amplified by heterogeneity, imprecision, and low-to-moderate GRADE certainty. Before routine clinical adoption, future multicenter prospective studies should use prespecified thresholds, standardized image acquisition and reporting, independent external validation, calibration assessment, and evaluations of whether AI-informed decisions improve patient outcomes. Until such evidence is available, AI-based VETC prediction should be used as investigational support within multidisciplinary assessment rather than as a standalone basis for management.

## Supplementary material

10.2196/90931 Multimedia Appendix 1 Supplementary tables.

10.2196/90931 Checklist 1 PRISMA-DTA checklist.

10.2196/90931 Checklist 2 CHARMS checklist.

10.2196/90931 Checklist 3 PRISMA checklist for abstracts.

10.2196/90931 Checklist 4 PRISMA-S checklist.

## Abbreviations

[^18^F]FDG PET/CT: 2-[¹⁸F]fluoro-2-deoxy-D-glucose positron emission tomography or computed tomography
AI: artificial intelligence
AUC: area under the curve
CECT: contrast-enhanced computed tomography
CEMRI: contrast-enhanced magnetic resonance imaging
CEUS: contrast-enhanced ultrasound
CHARMS: Checklist for Critical Appraisal and Data Extraction for Systematic Reviews of Prediction Modelling Studies
CT: computed tomography
DOR: diagnostic odds ratio
FN: false negative
FP: false positive
GRADE: Grading of Recommendations, Assessment, Development, and Evaluation
HCC: hepatocellular carcinoma
HKSJ: Hartung-Knapp-Sidik-Jonkman
HR: hazard ratio
MLP: multilayer perceptron
MRI: magnetic resonance imaging
PI: prediction interval
PITROS: population, index test, target condition, reference standard, outcomes, and setting
PRISMA: Preferred Reporting Items for Systematic Reviews and Meta-Analyses
PRISMA-DTA: Preferred Reporting Items for Systematic Reviews and Meta-Analyses for Diagnostic Test Accuracy
PRISMA-S: Preferred Reporting Items for Systematic Reviews and Meta-Analyses literature search extension
PROBAST: Prediction model Risk Of Bias Assessment Tool
REML: restricted maximum likelihood
RF: random forest
TN: true negative
TP: true positive
VETC: vessels encapsulating tumor cluster
