# Supplementary material for: Radiomics-Based AI for the Diagnosis and Prognosis of Vessels Encapsulating Tumor Clusters in Hepatocellular Carcinoma: Systematic Review and Meta-Analysis
Source: J Med Internet Res. 2026 Jul 2;28:e90931. doi: 10.2196/90931 (PMC13328951; doi:10.2196/90931)
Supplement: Multimedia Appendix 1 [file jmir-v28-e90931-s001.docx]

**Table of contents**

[Table S1 Search strategy in PubMed, Embase, Cochrane and Web of Science. 2](#_Toc233105490)

[Table S2 Risk of bias assessment (PROBAST+AI) model development. 8](#_Toc233105491)

[Table S3 Risk of bias assessment (PROBAST+AI) model evaluation based on seven domains 11](#_Toc233105492)

[Table S4 GRADE scoring assessments in all of the pooled outcomes. 14](#_Toc233105493)

[Table S5 Technical aspects of included studies for MRI. 16](#_Toc233105494)

[Table S6 Technical aspects of included studies for CT 18](#_Toc233105495)

[Table S7 Technical aspects of included studies for PET/ CT. 19](#_Toc233105496)

[Table S8 Technical aspects of included studies for US. 20](#_Toc233105497)

### **Table S1.** Search strategy in PubMed, Embase, Cochrane and Web of Science.

| Database | Search strategy | Filters and Limits | Number of Studies |
| --- | --- | --- | --- |
| PubMed | ("Radiomics"[Mesh] OR "Artificial Intelligence"[Mesh] OR "Machine Learning"[Mesh] OR "Deep Learning"[Mesh] OR "Neural Networks, Computer"[Mesh] OR "Image Processing, Computer-Assisted"[Mesh] OR "Diagnosis, Computer-Assisted"[Mesh] OR "Image Interpretation, Computer-Assisted"[Mesh] OR "Pattern Recognition, Automated"[Mesh] OR "Support Vector Machine"[Mesh] OR "Algorithms"[Mesh] OR "Data Mining"[Mesh] OR "Artificial Intelligence"[tiab] OR "AI"[tiab] OR "Machine Learning"[tiab] OR "Deep Learning"[tiab] OR "Machine Intelligence"[tiab] OR "Radiomic*"[tiab] OR "radiomic feature*"[tiab] OR "radiomic analysis"[tiab] OR "radiomic signature*"[tiab] OR "radiomic model*"[tiab] OR "radiomic score*"[tiab] OR "radiomics-based"[tiab] OR "image analysis"[tiab] OR "image-based biomarker*"[tiab] OR "imaging biomarker*"[tiab] OR "predictive model*"[tiab] OR "prediction model*"[tiab] OR "algorithm*"[tiab] OR "data mining"[tiab] OR "neural network*"[tiab] OR "convolutional neural network*"[tiab] OR "CNN"[tiab] OR "support vector machine*"[tiab] OR "SVM"[tiab] OR "random forest*"[tiab] OR "XGBoost"[tiab] OR "gradient boosting"[tiab] OR "LASSO"[tiab] OR "decision tree*"[tiab] OR "transfer learning"[tiab] OR "feature extraction"[tiab] OR "feature selection"[tiab] OR "nomogram*"[tiab] OR "fractal analysis"[tiab] OR "texture analysis"[tiab] OR "texture feature*"[tiab] OR "quantitative imaging"[tiab] OR "computer-aided"[tiab] OR "computer aided"[tiab] OR "computer-assisted"[tiab] OR "computer assisted"[tiab] OR "habitat imaging"[tiab] OR "delta radiomics"[tiab] OR "ResNet"[tiab] OR "U-Net"[tiab] OR "VGG"[tiab] OR "ensemble learning"[tiab] OR "AutoML"[tiab] OR "pyradiomics"[tiab]) AND ("Liver Neoplasms"[Mesh] OR "Carcinoma, Hepatocellular"[Mesh] OR "Hepatocellular carcinoma"[tiab] OR "HCC"[tiab] OR "liver cancer*"[tiab] OR "liver tumor*"[tiab] OR "liver tumour*"[tiab] OR "hepatic carcinoma"[tiab] OR "liver carcinoma"[tiab] OR "liver mass*"[tiab] OR "hepatobiliary cancer*"[tiab] OR "liver lesion*"[tiab] OR "hepatic neoplasm*"[tiab] OR "hepatic tumor*"[tiab] OR "hepatic tumour*"[tiab] OR "hepatic cancer"[tiab] OR "hepatic mass*"[tiab] OR "hepatic lesion*"[tiab] OR "primary liver cancer"[tiab] OR "liver malignancy"[tiab] OR "hepatic malignancy"[tiab]) AND ("Vessels Encapsulating Tumor Clusters"[tiab] OR "VETC"[tiab] OR "vessels encapsulate tumor cluster*"[tiab] OR "vessels encapsulating tumor cluster*"[tiab] OR "microvascular invasion"[tiab] OR "MVI"[tiab] OR "macrovascular invasion"[tiab] OR "macroscopic vascular invasion"[tiab] OR "microscopic vascular invasion"[tiab] OR "microvessel invasion"[tiab] OR "portal vein invasion"[tiab] OR "portal vein tumor thrombus"[tiab] OR "portal vein tumour thrombus"[tiab] OR "PVTT"[tiab]) AND ("Magnetic Resonance Imaging"[Mesh] OR "Tomography, X-Ray Computed"[Mesh] OR "Positron Emission Tomography Computed Tomography"[Mesh] OR "Positron-Emission Tomography"[Mesh] OR "Ultrasonography"[Mesh] OR "Contrast Media"[Mesh] OR "Multidetector Computed Tomography"[Mesh] OR "MRI"[tiab] OR "magnetic resonance"[tiab] OR "MR imaging"[tiab] OR "CEMRI"[tiab] OR "contrast-enhanced MRI"[tiab] OR "contrast enhanced MRI"[tiab] OR "Gd-EOB-DTPA"[tiab] OR "gadoxetic acid"[tiab] OR "gadoxetate"[tiab] OR "hepatobiliary phase"[tiab] OR "diffusion-weighted"[tiab] OR "DWI"[tiab] OR "computed tomography"[tiab] OR "CT scan*"[tiab] OR "CECT"[tiab] OR "contrast-enhanced CT"[tiab] OR "contrast enhanced CT"[tiab] OR "multiphasic CT"[tiab] OR "multiphase CT"[tiab] OR "dual-energy CT"[tiab] OR "ultrasound"[tiab] OR "ultrasonography"[tiab] OR "CEUS"[tiab] OR "contrast-enhanced ultrasound"[tiab] OR "contrast enhanced ultrasound"[tiab] OR "PET"[tiab] OR "PET/CT"[tiab] OR "PET-CT"[tiab] OR "FDG"[tiab] OR "fluorodeoxyglucose"[tiab] OR "18F-FDG"[tiab] OR "[18F]FDG"[tiab] OR "positron emission tomography"[tiab]) | No restrictions applied regarding date range, language, or publication type. | 683 |
| Embase | ('radiomics'/exp OR 'artificial intelligence'/exp OR 'machine learning'/exp OR 'deep learning'/exp OR 'artificial neural network'/exp OR 'convolutional neural network'/exp OR 'computer assisted diagnosis'/exp OR 'image analysis'/exp OR 'image processing'/exp OR 'pattern recognition'/exp OR 'support vector machine'/exp OR 'algorithm'/exp OR 'data mining'/exp OR 'random forest'/exp OR 'nomogram'/exp OR 'artificial intelligence':ab,ti OR 'AI':ab,ti OR 'machine learning':ab,ti OR 'deep learning':ab,ti OR 'machine intelligence':ab,ti OR 'radiomic*':ab,ti OR 'radiomic feature*':ab,ti OR 'radiomic analysis':ab,ti OR 'radiomic signature*':ab,ti OR 'radiomic model*':ab,ti OR 'radiomic score*':ab,ti OR 'image analysis':ab,ti OR 'image-based biomarker*':ab,ti OR 'imaging biomarker*':ab,ti OR 'predictive model*':ab,ti OR 'prediction model*':ab,ti OR 'algorithm*':ab,ti OR 'data mining':ab,ti OR 'neural network*':ab,ti OR 'convolutional neural network*':ab,ti OR 'CNN':ab,ti OR 'support vector machine*':ab,ti OR 'SVM':ab,ti OR 'random forest*':ab,ti OR 'XGBoost':ab,ti OR 'gradient boosting':ab,ti OR 'LASSO':ab,ti OR 'decision tree*':ab,ti OR 'transfer learning':ab,ti OR 'feature extraction':ab,ti OR 'feature selection':ab,ti OR 'nomogram*':ab,ti OR 'fractal analysis':ab,ti OR 'texture analysis':ab,ti OR 'texture feature*':ab,ti OR 'quantitative imaging':ab,ti OR 'computer-aided':ab,ti OR 'computer aided':ab,ti OR 'computer-assisted':ab,ti OR 'habitat imaging':ab,ti OR 'delta radiomics':ab,ti OR 'ResNet':ab,ti OR 'U-Net':ab,ti OR 'VGG':ab,ti OR 'ensemble learning':ab,ti OR 'AutoML':ab,ti OR 'pyradiomics':ab,ti) AND ('liver tumor'/exp OR 'liver cell carcinoma'/exp OR 'liver cancer'/exp OR 'hepatocellular carcinoma':ab,ti OR 'HCC':ab,ti OR 'liver cancer*':ab,ti OR 'liver tumor*':ab,ti OR 'liver tumour*':ab,ti OR 'hepatic carcinoma':ab,ti OR 'liver carcinoma':ab,ti OR 'liver mass*':ab,ti OR 'hepatobiliary cancer*':ab,ti OR 'liver lesion*':ab,ti OR 'hepatic neoplasm*':ab,ti OR 'hepatic tumor*':ab,ti OR 'hepatic tumour*':ab,ti OR 'hepatic cancer':ab,ti OR 'hepatic mass*':ab,ti OR 'hepatic lesion*':ab,ti OR 'primary liver cancer':ab,ti OR 'liver malignancy':ab,ti OR 'hepatic malignancy':ab,ti) AND ('vessels encapsulate tumor cluster*':ab,ti OR 'vessels encapsulating tumor cluster*':ab,ti OR 'VETC':ab,ti OR 'microvascular invasion':ab,ti OR 'MVI':ab,ti OR 'macrovascular invasion':ab,ti OR 'macroscopic vascular invasion':ab,ti OR 'microscopic vascular invasion':ab,ti OR 'microvessel invasion':ab,ti OR 'portal vein invasion':ab,ti OR 'portal vein tumor thrombus':ab,ti OR 'portal vein tumour thrombus':ab,ti OR 'PVTT':ab,ti) AND ('nuclear magnetic resonance imaging'/exp OR 'computer assisted tomography'/exp OR 'positron emission tomography-computed tomography'/exp OR 'positron emission tomography'/exp OR 'echography'/exp OR 'contrast medium'/exp OR 'multidetector computed tomography'/exp OR 'MRI':ab,ti OR 'magnetic resonance':ab,ti OR 'MR imaging':ab,ti OR 'CEMRI':ab,ti OR 'contrast-enhanced MRI':ab,ti OR 'Gd-EOB-DTPA':ab,ti OR 'gadoxetic acid':ab,ti OR 'gadoxetate':ab,ti OR 'hepatobiliary phase':ab,ti OR 'diffusion-weighted':ab,ti OR 'DWI':ab,ti OR 'computed tomography':ab,ti OR 'CT scan*':ab,ti OR 'CECT':ab,ti OR 'contrast-enhanced CT':ab,ti OR 'multiphasic CT':ab,ti OR 'multiphase CT':ab,ti OR 'dual-energy CT':ab,ti OR 'ultrasound':ab,ti OR 'ultrasonography':ab,ti OR 'CEUS':ab,ti OR 'contrast-enhanced ultrasound':ab,ti OR 'PET':ab,ti OR 'PET/CT':ab,ti OR 'PET-CT':ab,ti OR 'FDG':ab,ti OR 'fluorodeoxyglucose':ab,ti OR '18F-FDG':ab,ti OR '[18F]FDG':ab,ti OR 'positron emission tomography':ab,ti) | No restrictions applied regarding date range, language, or publication type. | 1773 |
| Web of Science | TS=("Radiomics" OR "Artificial Intelligence" OR "AI" OR "Machine Learning" OR "Deep Learning" OR "Machine Intelligence" OR "Radiomic*" OR "radiomic feature*" OR "radiomic analysis" OR "radiomic signature*" OR "radiomic model*" OR "radiomic score*" OR "radiomics-based" OR "image analysis" OR "image-based biomarker*" OR "imaging biomarker*" OR "predictive model*" OR "prediction model*" OR "algorithm*" OR "data mining" OR "neural network*" OR "convolutional neural network*" OR "CNN" OR "support vector machine*" OR "SVM" OR "random forest*" OR "XGBoost" OR "gradient boosting" OR "LASSO" OR "decision tree*" OR "transfer learning" OR "feature extraction" OR "feature selection" OR "nomogram*" OR "fractal analysis" OR "texture analysis" OR "texture feature*" OR "quantitative imaging" OR "computer-aided" OR "computer aided" OR "computer-assisted" OR "computer assisted" OR "habitat imaging" OR "delta radiomics" OR "ResNet" OR "U-Net" OR "VGG" OR "ensemble learning" OR "AutoML" OR "pyradiomics") AND TS=("Hepatocellular carcinoma" OR "HCC" OR "liver cancer*" OR "liver tumor*" OR "liver tumour*" OR "hepatic carcinoma" OR "liver carcinoma" OR "liver mass*" OR "hepatobiliary cancer*" OR "liver lesion*" OR "hepatic neoplasm*" OR "hepatic tumor*" OR "hepatic tumour*" OR "hepatic cancer" OR "hepatic mass*" OR "hepatic lesion*" OR "primary liver cancer" OR "liver malignancy" OR "hepatic malignancy" OR "Liver Neoplasms" OR "Carcinoma Hepatocellular") AND TS=("Vessels Encapsulating Tumor Clusters" OR "VETC" OR "vessels encapsulate tumor cluster*" OR "vessels encapsulating tumor cluster*" OR "microvascular invasion" OR "MVI" OR "macrovascular invasion" OR "macroscopic vascular invasion" OR "microscopic vascular invasion" OR "microvessel invasion" OR "portal vein invasion" OR "portal vein tumor thrombus" OR "portal vein tumour thrombus" OR "PVTT") AND TS=("MRI" OR "magnetic resonance" OR "MR imaging" OR "CEMRI" OR "contrast-enhanced MRI" OR "contrast enhanced MRI" OR "Gd-EOB-DTPA" OR "gadoxetic acid" OR "gadoxetate" OR "hepatobiliary phase" OR "diffusion-weighted" OR "DWI" OR "computed tomography" OR "CT scan*" OR "CECT" OR "contrast-enhanced CT" OR "contrast enhanced CT" OR "multiphasic CT" OR "multiphase CT" OR "dual-energy CT" OR "ultrasound" OR "ultrasonography" OR "CEUS" OR "contrast-enhanced ultrasound" OR "contrast enhanced ultrasound" OR "PET" OR "PET/CT" OR "PET-CT" OR "FDG" OR "fluorodeoxyglucose" OR "18F-FDG" OR "positron emission tomography") | No restrictions applied regarding date range, language, or publication type. | 661 |
| Cochrane Library | ("Radiomics" OR "Artificial Intelligence" OR "AI" OR "Machine Learning" OR "Deep Learning" OR "Machine Intelligence" OR "Radiomic*" OR "radiomic feature*" OR "radiomic analysis" OR "radiomic signature*" OR "radiomic model*" OR "image analysis" OR "image-based biomarker*" OR "imaging biomarker*" OR "predictive model*" OR "prediction model*" OR "algorithm*" OR "data mining" OR "neural network*" OR "convolutional neural network*" OR "CNN" OR "support vector machine*" OR "SVM" OR "random forest*" OR "XGBoost" OR "gradient boosting" OR "LASSO" OR "decision tree*" OR "transfer learning" OR "feature extraction" OR "feature selection" OR "nomogram*" OR "fractal analysis" OR "texture analysis" OR "texture feature*" OR "quantitative imaging" OR "computer-aided" OR "computer-assisted" OR "habitat imaging" OR "delta radiomics"):ti,ab,kw AND ("Hepatocellular carcinoma" OR "HCC" OR "liver cancer*" OR "liver tumor*" OR "liver tumour*" OR "hepatic carcinoma" OR "liver carcinoma" OR "liver mass*" OR "hepatobiliary cancer*" OR "liver lesion*" OR "hepatic neoplasm*" OR "hepatic tumor*" OR "hepatic cancer" OR "hepatic mass*" OR "hepatic lesion*" OR "primary liver cancer" OR "liver malignancy" OR "hepatic malignancy" OR "Liver Neoplasms" OR "Carcinoma Hepatocellular"):ti,ab,kw AND ("Vessels Encapsulating Tumor Clusters" OR "VETC" OR "vessels encapsulate tumor cluster*" OR "vessels encapsulating tumor cluster*" OR "microvascular invasion" OR "MVI" OR "macrovascular invasion" OR "microscopic vascular invasion" OR "microvessel invasion" OR "portal vein invasion" OR "portal vein tumor thrombus" OR "PVTT"):ti,ab,kw AND ("MRI" OR "magnetic resonance" OR "MR imaging" OR "CEMRI" OR "contrast-enhanced MRI" OR "Gd-EOB-DTPA" OR "gadoxetic acid" OR "diffusion-weighted" OR "DWI" OR "computed tomography" OR "CT scan*" OR "CECT" OR "contrast-enhanced CT" OR "ultrasound" OR "ultrasonography" OR "CEUS" OR "contrast-enhanced ultrasound" OR "PET" OR "PET/CT" OR "PET-CT" OR "FDG" OR "positron emission tomography"):ti,ab,kw | No restrictions applied regarding date range, language, or publication type. | 21 |

### **Table S2.** Risk of bias assessment (PROBAST+AI) model development.

| Author, year | | Quality | | | | Applicability concerns | | | Overall judgement | |
| --- | --- | --- | --- | --- | --- | --- | --- | --- | --- | --- |
|  |  | Participants and data sources ^a^ | Predictors ^b^ | Outcome ^c^ | Analysis ^d^ | Participants and data sources ^e^ | Predictors ^f^ | Outcome ^g^ | Quality ^h^ | Applicability concerns ^i^ |
| X Dong et al. | 2024 | L | L | L | L | L | L | L | L | L |
| Q Qu et al. | 2024 | L | L | L | L | L | H | L | L | H |
| F Che et al. | 2025 | L | L | L | L | L | L | L | L | L |
| Matsuda et al. | 2025 | L | L | L | L | L | H | L | L | H |
| MT Gu et al. | 2025 | L | L | L | L | L | L | L | L | L |
| JW Yang et al. | 2024 | L | L | L | L | L | L | L | L | L |
| SQ Hu et al. | 2025 | L | L | L | L | L | L | L | L | L |
| JY Zhang et al. | 2024 | L | L | L | L | L | L | L | L | L |
| WX Xu et al. | 2024 | L | L | L | L | L | L | L | L | L |
| YM Zhao et al. | 2025 | L | L | L | L | L | L | L | L | L |
| JJ Wang et al. | 2025 | L | L | L | L | L | L | L | L | L |
| Chao Zhang et al. | 2024 | L | L | L | L | L | L | L | L | L |
| YX Yu et al. | 2021 | L | L | L | L | L | L | L | L | L |
| TJ Chu et al. | 2022 | L | L | L | H | L | L | L | H | L |
| WD Wang et al. | 2025 | L | L | L | L | L | L | L | L | L |

**Abbreviation:** PROBAST+AI, Prediction model Risk of Bias Assessment Tool + AI, L low; H high; U unclear.

**Footnote:** Signaling questions are rated as "yes" (Y), "probably yes" (PY), "probably no" (PN), "no" (N), "no information" (NI), and in some cases "not applicable" (NA). All signaling questions are phrased in such a way that "yes" or "probably yes" indicates a low risk of bias. Any signaling questions rated as "no" or "probably no" indicate a potential high risk of bias in that domain. If there are no "no" or "probably no" ratings, but "no information" (NI) is present, the risk of bias in that domain is classified as unclear.

**a. Participants and data sources**

1.1 Were appropriate data sources used?

1.2 Was an appropriate study design used?

1.3 Did the in- and exclusions of study participants result in a representative dataset?

**b. Predictors**

2.1 Were predictors defined and assessed in a similar way for all participants?

2.2 Was any pre-processing of predictors similar for all participants?

2.3 Were the predictors included in the model available at the time the model was intended to be used?

**c. Outcome**

3.1 Were outcomes defined and assessed appropriately?

3.2 Were outcomes defined and assessed in a similar way for all participants?

3.3 Were outcome assessments made without use or knowledge of predictor data?

3.4 Was the time interval between predictor assessment and outcome assessment appropriate?

**d. Analysis**

4.1 Was there evidence that the sample size was reasonable?

4.2 Were continuous and categorical predictors handled appropriately?

4.3 Were participants with missing or censored data handled appropriately in the analysis?

4.4 If methods to address class imbalance were used, was the model or the model predictions recalibrated?

4.5 Were methods used to address potential model overfitting?

**e. Participants and data sources**

Concern that the (data of the) included participants do not match the review question or the assessor’s intended use of the prediction model.

f. **Predictors**

Concern that the definition, pre-processing, assessment, or timing of assessment of the predictors in the model do not match the review question or the assessor’s intended use.

g. **Outcome**

Concern that the outcome, its definition, assessment, or timing of assessment do not match the review question or the assessor’s intended use.

**h. Quality**

Low risk: If all four domains were rated low concern regarding quality.

High risk: If at least one domain was rated high concern regarding quality.

Unclear: If at least one domain was rated unclear concern regarding quality and no domains were rated high concern.

**i. Applicability concerns**

Low risk: If all three domains were rated low concern for applicability.

High risk: If at least one domain was rated high concern for applicability.

Unclear: If at least one domain was rated unclear concern for applicability and no domains were rated high concern.

### **Table S3.** Risk of bias assessment (PROBAST+AI) model evaluation based on seven domains

| Author, year | | Risk of bias | | | | Applicability concerns | | | Overall judgement | |
| --- | --- | --- | --- | --- | --- | --- | --- | --- | --- | --- |
|  |  | Participants and data sources ^a^ | Predictors ^b^ | Outcome ^c^ | Analysis ^d^ | Participants and data sources ^e^ | Predictors ^f^ | Outcome ^g^ | Risk of bias ^h^ | Applicability concerns ^i^ |
| X Dong et al. | 2024 | L | L | U | L | L | L | L | U | L |
| Q Qu et al. | 2024 | L | L | L | L | L | L | L | L | L |
| F Che et al. | 2025 | L | L | L | L | L | L | L | L | L |
| Matsuda et al. | 2025 | L | L | U | L | L | L | L | U | L |
| MT Gu et al. | 2025 | L | L | L | L | L | L | L | L | L |
| JW Yang et al. | 2024 | L | L | L | L | L | L | L | L | L |
| SQ Hu et al. | 2025 | L | L | U | H | L | L | L | H | L |
| JY Zhang et al. | 2024 | L | L | L | L | L | L | L | L | L |
| WX Xu et al. | 2024 | L | L | L | H | L | L | L | H | L |
| YM Zhao et al. | 2025 | L | L | L | L | L | L | L | L | L |
| JJ Wang et al. | 2025 | L | L | L | L | L | L | L | L | L |
| Chao Zhang et al. | 2024 | L | L | L | L | L | L | L | L | L |
| YX Yu et al. | 2021 | L | L | L | L | L | L | L | L | L |
| TJ Chu et al. | 2022 | L | L | U | H | L | L | L | H | L |
| WD Wang et al. | 2025 | L | L | L | U | L | L | L | U | L |

**Abbreviation:** PROBAST+AI, Prediction model Risk of Bias Assessment Tool + AI, L low; H high; U unclear.

**Footnote:** Signaling questions are rated as "yes" (Y), "probably yes" (PY), "probably no" (PN), "no" (N), "no information" (NI), and in some cases "not applicable" (NA). All signaling questions are phrased in such a way that "yes" or "probably yes" indicates a low risk of bias. Any signaling questions rated as "no" or "probably no" indicate a potential high risk of bias in that domain. If there are no "no" or "probably no" ratings, but "no information" (NI) is present, the risk of bias in that domain is classified as unclear.

**a. Participants and data sources**

1.1 Were appropriate data sources used?

1.2 Was an appropriate study design used?

1.3 Did the in- and exclusions of study participants result in a representative dataset?

**b. Predictors**

2.1 Were predictors defined and assessed in a similar way for all participants?

2.2 Was any pre-processing of predictors similar for all participants?

2.3 Were the predictors included in the model available at the time the model was intended to be used?

**c. Outcome**

3.1 Were outcomes defined and assessed appropriately?

3.2 Were outcomes defined and assessed in a similar way for all participants?

3.3 Were outcome assessments made without use or knowledge of predictor data?

3.4 Was the time interval between predictor assessment and outcome assessment appropriate?

**d. Analysis**

4.1 Was model evaluation based on only apparent performance avoided?

4.2 Was there evidence that the sample size was reasonable?

4.3 Were participants with missing or censored data handled appropriately in the analysis?

4.4 If methods to address class imbalance were used, was the evaluation done in a dataset without imbalance correction?

4.5 If data splitting was done to create training and test datasets, was there evidence that data leakage was avoided?

4.6 If resampling methods were used to evaluate model performance, were all model development steps replicated in the resampling process?

4.7 Was the predictive performance of the model evaluated appropriately, e.g., calibration, discrimination, and net benefit?

**e. Participants and data sources**

Concern that the (data of the) included participants do not match the review question or the assessor’s intended use of the prediction model.

f. **Predictors**

Concern that the definition, pre-processing, assessment, or timing of assessment of the predictors in the model do not match the review question or the assessor’s intended use.

g. **Outcome**

Concern that the outcome, its definition, assessment, or timing of assessment do not match the review question or the assessor’s intended use.

**h. Risk of bias**

Low risk: If all four domains were rated low risk of bias.

High risk: If at least one domain was rated high risk of bias.

Unclear: If at least one domain was rated unclear risk of bias and no domains were rated high risk of bias.

**i. Applicability concerns**

Low risk: If all three domains were rated low concern for applicability.

High risk: If at least one domain was rated high concern for applicability.

Unclear: If at least one domain was rated unclear concern for applicability and no domains were rated high concern.

### **Table S4.** GRADE scoring assessments in all of the pooled outcomes.

| Dataset | Outcome | Risk of Bias^a^ | Inconsistency^b^ | Indirectness^c^ | Imprecision^d^ | Publication Bias^e^ | Total Downgrade | Final Rating |
| --- | --- | --- | --- | --- | --- | --- | --- | --- |
| CEMRI-based AI | Sensitivity | 1 | 0 | 0 | 1 | 0 | 2 | low |
|  | Specificity | 1 | 0 | 0 | 1 | 0 | 2 | low |
| CECT-based AI | Sensitivity | 0 | 0 | 0 | 1 | 0 | 1 | Moderate |
|  | Specificity | 0 | 0 | 0 | 1 | 0 | 1 | Moderate |
| [^18^F] FDG PET/CT-based AI | Sensitivity | 1 | 0 | 0 | 1 | 0 | 2 | low |
|  | Specificity | 1 | 0 | 0 | 1 | 0 | 2 | low |
| CEUS-based AI | Sensitivity | 1 | 0 | 0 | 1 | 0 | 2 | low |
|  | Specificity | 1 | 0 | 0 | 1 | 0 | 2 | low |

a. Risk of Bias

Assessed using tools like PROSBAST+AI to evaluate study design and methodology.

Downgrade by 1 level if at least one study has a high risk of bias.

b. Inconsistency

Measured using I² statistics to assess heterogeneity across studies.

Downgrade by 1 level if I² > 50% and the source of heterogeneity cannot be explained.

If the heterogeneity is identified (e.g., through meta-regression), no downgrade is applied.

c. Indirectness

Evaluates whether study populations, interventions, or outcomes differ from the target research question.

Patient Indirectness: Downgrade if the included population significantly deviates from the target population (e.g., specific subgroups).

Outcome Indirectness: Downgrade if inconsistent outcome measures are used, such as estimating sensitivity/specificity indirectly from ROC-based Youden Index.

d. Imprecision

Assessed based on confidence intervals (CIs) and sample size sufficiency.

For sensitivity/specificity: Downgrade if 95% CI width > 0.20 or if CIs cross clinical thresholds (e.g., 0.80).

Sample size threshold: Total sample < 500 or positive/negative cases < 100.

e. Publication Bias

Evaluated using Deek’s Funnel Plot or similar tools.

Downgrade by 1 level if the funnel plot shows significant asymmetry or if P-value < 0.05 in Deek’s tests.

### **Table S5.** Technical aspects of included studies for MRI.

| Author | Year | MRI field strength (Manufacturer-Scanner Modality) | Evaluation Time | Regions of interest | MRI sequence | Enhancement phase | Contrast-enhanced agents |
| --- | --- | --- | --- | --- | --- | --- | --- |
| Matsuda et al. | 2025 | 1.5 T (GE Healthcare-Signa ES, Signa HD, Sign HDxt, Signa Excite, Optima MR450W) and 3.0T (GE Healthcare-Discovery MR750, Signa Pioneer,) | Pre-operative | Manually | NA | AP, HBP | Gd-EOB-DTPA |
| MT Gu et al. | 2025 | 1.5 T (GE Healthcare-Optima MR360) and 3.0T (GE Healthcare-Discovery MR750; Philips Medical Systems Nederland B.V-Philips Ingenia) | Pre-operative | Manually | T1WI, T2WI, DWI | AP, PVP, HBP, DP | Gd-BOPTA |
| F Che et al | 2025 | 3.0 T (GE Healthcare- Discovery MR 750, SIGNA™ Architect, SIGNA™ Premier, GE Discovery MR 750; Siemens Healthineers- MAGNETOM Skyra) | Pre-operative | Manually | T1WI, T2WI, DWI | PCP, L-AP, PVP, TP, HBP | Gd-EOB-DTPA |
| X Dong et al. | 2023 | 1.5 T (GE Healthcare-Signa Twinspeed) and 3.0 T (GE Healthcare-Discover MR750) | Pre-operative | Semi-automatic | T1WI, T2WI, DWI | AP, PVP, DP | Gd-DTPA |
| JY Zhang et al. | 2024 | 3.0 T (Philips Healthcare- Intera Achieva) | Pre-operative | Manually | FSDE-T1WI, FS T2WI | AP, PVP, HBP | Gd-EOB-DTPA |
| Yang et al. JY Yang | 2024 | 1.5T (GE Healthcare-Signa Twinspeed) and 3.0 T (GE Healthcare- SIGNA HDx, Discover MR750) | Pre-operative | Manually | BHA-T1WI, DCE-T1WI, T2WI, DWI | AP, PVP, DP | NA |
| Qu et al. | 2023 | 3.0 T (Philips Healthcre- Intera Achieva) | Pre-operative | Manually | T1WI, T2WI, DWI, DCES | AP, PVP, TP, HBP | Gd-EOB-DTPA |
| Chu et al. | 2022 | 3.0 T (GE Healthcare- Discovery MR750; Philips Medical Systems- Ingenia 3.0 T, Achieva 3.0 T) | Pre-operative | Manually | T1WI | L-AP, PVP, HBP | Gd-EOB-DTPA |
| Yu et al. | 2021 | 3.0 T (Siemens Healthcare- Magnetom Verio) | Pre-operative | Manually | 3D-FS-VIBE-T1WI | AP, PVP, TP, HBP | Gd-EOB-DTPA |
| Wang et al. | 2025 | 3.0 T (Siemens-Magnetom Skyra 3.0 T; GE Healthcare- SIGNA Premier 3.0 T; Philips- Achieva 3.0 T) | Pre-operative | Manually | T1WI, FS-T2WI, DWI, MCES | AP, PVP, HBP | Gd‑EOB‑DTPA |

AP arterial phase; L-AP late arterial phase; PVP portal venous phase; PCP pre-contrast phase; HBP hepatobiliary phase; TP transitional phase; DP delayed phase; T1WI T1-weighted imaging; BHA breath-hold axial; T2WI T2-weighted imaging; DWI diffusion-weighted imaging; MCES multiphase contrast-enhanced scanning; DCE dynamic contrast-enhanced; VIBE volumetric interpolated breath-hold examination; FS fat-suppressed; FSDE fast spoiled dual-echo; Gd‑EOB‑DTPA gadolinium-ethoxybenzyl-diethylenetriamine pentaacetic acid; Gd-BOPTA gadolinium-benzyloxypropionic acid-tetraacetic acid; Gd-DTPA gadolinium-diethylenetriamine pentaacetic acid.

### **Table S6.** Technical aspects of included studies for CT

| Author | Year | Manufacturer (Scanner Modality) | Evaluation Time | Regions of interest | Enhancement phase | Contrast-enhanced agents (Dose) |
| --- | --- | --- | --- | --- | --- | --- |
| Zhao et al. | 2025 | GE Healthcare (GE Revolution CT, GE Discovery 64-layer helical CT) and Siemens (Siemens Dual-Source CT, Siemens 128-layer CT devices) | Pre-operative | Manually | AP, PVP, DP | Iodohexol (1.0–1.5 mL/kg) |
| Chao Zhang et al. | 2024 | GE Healthcare (256-section multidetector CT scanner) and Siemens (128-section multidetector CT scanner) | Pre-operative | Semi-automatic | AP, PVP, DP | Omnipaque (1.5mL/kg) |
| YY Wei et al. | 2025 | Siemens (Siemens Sensation 64-slice spiral CT) and GE Healthcare (GE Discovery CT 750 HD spiral CT) | Pre-operative | Manually | AP, VP, PP | Iopromide (1.5 mL/kg) |
| WD Wang et al. | 2025 | GE Healthcare (Discovery CT750hd) | Pre-operative | Manually | AP | Ioversol (1.0 mL/kg) |

AP arterial phase; PVP portal venous phase; DP delayed phase.

### **Table S7.** Technical aspects of included studies for PET/ CT.

| Author | Year | Manufacturer-Scanner Modality | Evaluation Time | Regions of interest (ROI) | Radiotracer | Dose |
| --- | --- | --- | --- | --- | --- | --- |
| Hu et al. | 2025 | GE Healthcare-Discovery Elite scanner and Siemens Healthcare- Biograph 64 PET/CT scanner | Pre-operative | Manually | [^18^F]FDG | 3.70–5.55 MBq/kg |

MBq/Kg megabecquerel per kilogram; FDG fluorodeoxyglucose; PET/ CT positron emission tomography / computed tomography.

### **Table S8.** Technical aspects of included studies for US.

| Author | Year | Manufacturer (Scanner Modality) | Evaluation Time | Regions of interest (ROI) | Ultrasound sequence | Contrast-enhanced agents |
| --- | --- | --- | --- | --- | --- | --- |
| WX Xu et al. | 2024 | Canon (Aplio, Aixplorer, and e-saote) | Pre-operative | Manually | AP, PVP, DP | SonoVue |

AP arterial phase; PVP portal venous phase; DP delayed phase.
